# Neuroretinitis With Severe Macular Edema in Dual Infection: Challenges in Management

**DOI:** 10.7759/cureus.58444

**Published:** 2024-04-17

**Authors:** Luqmanhaqim Aminuddin, Wan-Hazabbah Wan Hitam, Embong Zunaina, Shahidatul-Adha Mohamad

**Affiliations:** 1 Department of Ophthalmology and Visual Science, School of Medical Sciences, Universiti Sains Malaysia, Kubang Kerian, MYS; 2 Department of Ophthalmology and Visual Science, School of Medical Sciences, Universiti Sains Malaysia, Kota Bharu, MYS

**Keywords:** herpes simplex virus type 1, ocular toxoplasma, intravitreal ranibizumab, macular oedema, neuroretinitis

## Abstract

Neuroretinitis is a potentially vision-threatening condition distinguished by swelling of the optic disc followed by the emergence of a macular star pattern. The majority of these clinical observations are typically linked to infections caused by bacteria, parasites, or viruses. We report a case of dual infections in neuroretinitis complicated with severe macular edema.

A 49-year-old lady presented with sudden onset left eye blurring of vision of one-week duration. Visual acuity was 6/6 in the right eye and 6/60 in the left eye. There was a left positive relative afferent pupillary defect with impaired optic nerve functions. A fundoscopy of the left eye showed optic disc swelling with a macular star. The right optic disc was also swollen. Vasculitis changes were observed in both posterior poles. The ocular coherence tomography of the left eye revealed the existence of macular edema, subretinal fluids, and an epiretinal membrane that extended from the optic disc to the fovea. Serological examinations were positive for toxoplasma and herpes simplex virus type 1. The patient was started on oral azithromycin, oral acyclovir, and oral corticosteroids. Left macular edema persisted despite the treatment. The patient was given a trial of a single injection of intravitreal ranibizumab. A remarkable reduction of subretinal fluids was seen post-intravitreal injection and continuation of medications. Intravitreal ranibizumab has shown significant outcomes in neuroretinitis with severe macula edema.

## Introduction

Neuroretinitis is a clinical entity that can cause severe, painless visual loss. It is characterized by inflammation of the optic nerve and the retina, clinically represented by the presence of optic disc swelling and macular star. Many a time, the cause is an infection, in which around two-thirds of the cases are due to cat scratch disease [1]. However, other infections can cause neuroretintis, especially in tropical countries, namely, toxoplasma parasites, tuberculosis, and herpes simplex viruses. Neuroretinitis may lead to severe macular edema that leads to management challenges. We report a case of dual infections in neuroretinitis complicated with severe macular edema that responds to intravitreal ranibizumab injection.

## Case presentation

A healthy 49-year-old woman presented with a blurring of vision in the left eye (LE) for a one-week duration. It involved the central part of her LE vision. There was no history of eye pain, redness, swelling, or discharge. The patient also did not experience any fever, headache, nausea, or vomiting. She denied a history of cat scratches and did not have any cats at home. She had good premorbid vision without glasses.

**Figure 1:**
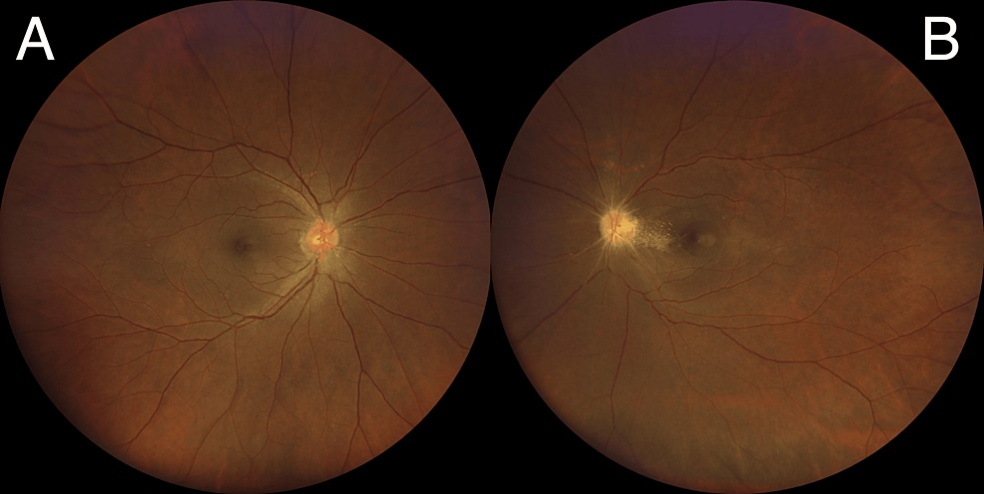
Fundus photos showing a bilaterally swollen optic disc with retinal vessel vasculitis changes (A & B); presence of partial macular star pattern in the left eye (B)

**Figure 2:**
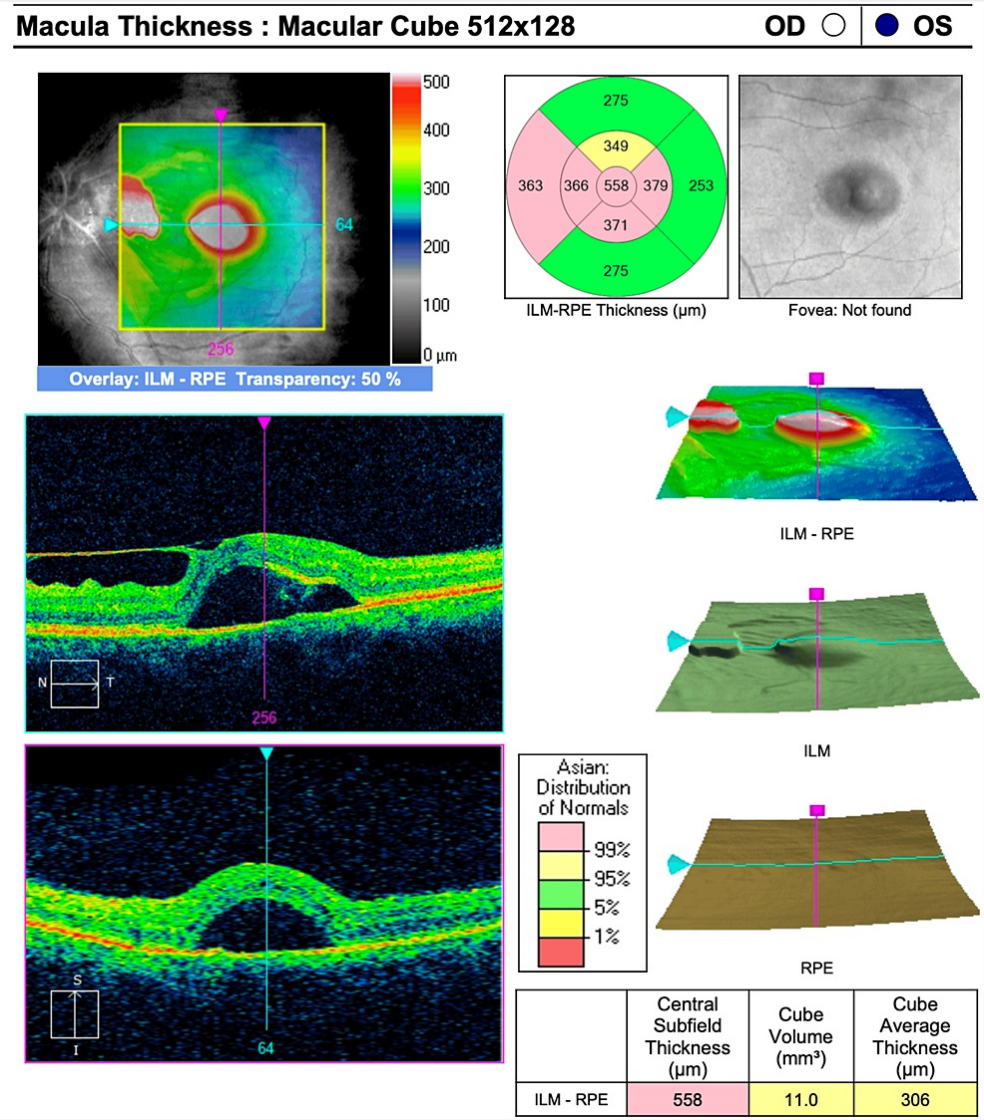
OCT macula of the left eye showed subretinal fluids at the center of the macula; the epiretinal membrane is extended from the optic disc to the fovea OCT: ocular coherence tomography

On examination, the best corrected visual acuity was 6/6 in the right eye (RE) and 6/60 in the LE. There was a positive left relative afferent pupillary defect with reduced optic nerve functions including light brightness, red saturation, and color vision. Her extraocular muscle movements were normal in both eyes. Both anterior segments were unremarkable with normal intraocular pressure. Fundoscopy of the LE showed optic disc swelling with hard exudates arranged in a partial macular star pattern. The right optic disc was also swollen. Vasculitis changes were observed in both posterior poles (Figure 1). Absence of any choroiditis or retinitis bilaterally. Her systemic examinations were unremarkable. The patient's blood pressure was within normal. Her cardiovascular and respiratory system examinations were also normal. There were no signs of neurological deficits or cranial nerve palsies. The ocular coherence tomography (OCT) of the LE revealed the existence of macular edema, subretinal fluids, and an epiretinal membrane that extends from the optic disc to the fovea (Figure 2). RE OCT was unremarkable.

The complete blood analysis revealed normal results. The erythrocyte sedimentation rate and C-reactive protein levels were within the normal range. The Mantoux test showed a normal measurement of 8 mm. The serological investigations revealed the presence of immunoglobulin G (IgG) antibodies for toxoplasma (2603 IU/ml) and herpes simplex virus type 1 (HSV-1). The serological test for *Bartonella henselae* showed a negative result. The results of the liver and kidney function tests were within the normal range. A contrast-enhanced CT scan of the brain revealed no signs of abnormal tissue growth or enhancement.

The patient was diagnosed with bilateral neuroretinitis secondary to dual infection (toxoplasmosis and HSV-1) with LE severe macular edema. She was started on oral azithromycin 500 mg daily for toxoplasmosis and acyclovir 400 mg twice a day for HSV-1 infections. A regimen of oral prednisolone was also initiated at a dosage of 60 mg per day, with a gradual decrease of 10mg per week for macular edema.

**Figure 3:**
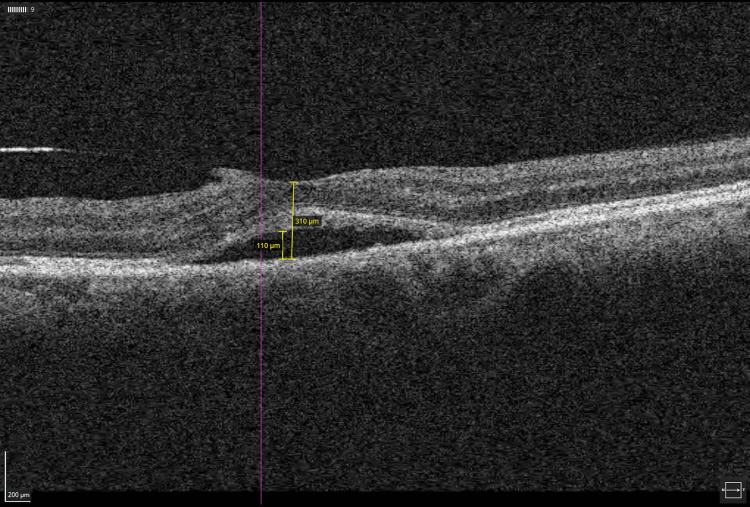
OCT macula of the left eye showed a significant reduction in subretinal fluids, and central macular thickness was seen at two weeks post-intravitreal ranibizumab injection OCT: ocular coherence tomography

The treatment for two weeks did not result in any improvement in the LE macular edema. She underwent a trial of a single intravitreal ranibizumab injection to her LE. The administration of oral azithromycin, acyclovir, and a tapering dosage of prednisolone was maintained. A subsequent review showed remarkable improvement in terms of visual acuity to 6/24 and a reduction of subretinal fluids in OCT two weeks after the intravitreal injection (Figure 3). Complete resolution of macular edema was seen after one month.

## Discussion

Neuroretinitis is a specific form of optic nerve disorder characterized by inflammation of the optic disc, accompanied by the appearance of hard exudates around both the optic nerve and macula [2]. The pathophysiology of neuroretinitis involves several processes, including increased permeability of disc blood vessels, inflammation of the optic disc blood vessels, and leakage of fluid into the adjacent peripapillary retina. Furthermore, neuroretinitis can be triggered by infectious diseases affecting the optic disc, post-viral or autoimmune responses, or unidentified idiopathic factors [3]. Most patients exhibit either unilateral or bilateral vision impairment. Asymmetrical involvement may manifest with a positive relative afferent pupil defect (RAPD) and decreased optic nerve function. The most frequently reported visual field defect by patients is centrocecal scotoma [4]. Our patient presented with diffuse central visual field defect due to severe macular edema.

Neuroretinitis investigations are performed to rule out the possibility of infectious causes. Infections play a significant role in the etiology of neuroretinitis. *Bartonella henselae*, the organism that is responsible for the cat-scratch disease, is commonly recognized as a frequent infectious cause of neuroretinitis [5,6]. Neuroretinitis has also been related to other infectious organisms, including *Toxoplasma gondii*, herpes simplex virus, and leptospira [7-9]. This patient had positive serology for toxoplasma and HSV-1. Oral azithromycin proves to be an effective treatment for ocular toxoplasmosis [10]. Azithromycin is advantageous for its once-daily dosing and lower incidence of adverse effects [11]. There is no standard treatment regime for HSV-related neuroretinitis. The patient was started with oral azithromycin and acyclovir. Lazaro-Rodriguez et al. reported isolated HSV-1 infection correlates with the presence of submacular fluids, and this condition has been successfully managed using a combination of antiviral agents and systemic corticosteroids [8]. A combination of antibiotics and corticosteroid treatment was associated with better visual outcomes, suggesting a potential role for corticosteroids in improving visual prognosis in certain ocular conditions [12].

Severe macular edema is rare in neuroretinitis, and its presence often complicates management. The presence of macular edema in neuroretinitis primarily contributes to the decline in central vision. Therefore, improvements in vision often coincide with the resolution of macular edema [13]. Corticosteroids may be administered orally, intravenously, or via intravitreal injection, depending on the severity of the condition and the patient's overall health status. The efficacy of intravitreal anti-vascular endothelial growth factor (anti-VEGF) agents in treating infectious neuroretinitis with macular edema has not been established. Anti-VEGF drugs like ranibizumab work by inhibiting the action of VEGF, binding directly to it, and preventing its interaction with receptors. This mechanism blocks downstream signaling pathways responsible for abnormal blood vessel growth and increased vascular permeability in the retina [14]. As a result, ranibizumab helps reduce vascular leakage and leads to the resolution of macular edema. However, the decision to use intravitreal ranibizumab should be made on a case-by-case basis after a thorough evaluation by a retina specialist. Our patient underwent a trial of a single intravitreal ranibizumab injection due to subretinal fluids at the macula. A reduction in subretinal fluid height was observed as a result.

## Conclusions

Neuroretinitis caused by a concurrent infection of toxoplasma and HSV-1 is uncommon. An optimal treatment typically involves the use of both antibiotics and antiviral medications. Macular edema is a recognized complication of neuroretinitis that may necessitate steroid administration, either orally, intravenously, or via intravitreal injection, depending on the severity of the condition. In cases of neuroretinitis with severe macular edema, intravitreal anti-VEGF agents represent a viable treatment option.
